# A Coupled Equilibrium Shift Mechanism in Calmodulin-Mediated Signal Transduction

**DOI:** 10.1016/j.str.2008.02.017

**Published:** 2008-05-07

**Authors:** Jörg Gsponer, John Christodoulou, Andrea Cavalli, Jennifer M. Bui, Barbara Richter, Christopher M. Dobson, Michele Vendruscolo

**Affiliations:** 1 Department of Chemistry, University of Cambridge, Lensfield Road, Cambridge CB2 1EW, United Kingdom

**Keywords:** PROTEINS, SIGNALING

## Abstract

We used nuclear magnetic resonance data to determine ensembles of conformations representing the structure and dynamics of calmodulin (CaM) in the calcium-bound state (Ca^2+^-CaM) and in the state bound to myosin light chain kinase (CaM-MLCK). These ensembles reveal that the Ca^2+^-CaM state includes a range of structures similar to those present when CaM is bound to MLCK. Detailed analysis of the ensembles demonstrates that correlated motions within the Ca^2+^-CaM state direct the structural fluctuations toward complex-like substates. This phenomenon enables initial ligation of MLCK at the C-terminal domain of CaM and induces a population shift among the substates accessible to the N-terminal domain, thus giving rise to the cooperativity associated with binding. Based on these results and the combination of modern free energy landscape theory with classical allostery models, we suggest that a coupled equilibrium shift mechanism controls the efficient binding of CaM to a wide range of ligands.

## Introduction

**Figure 1:**
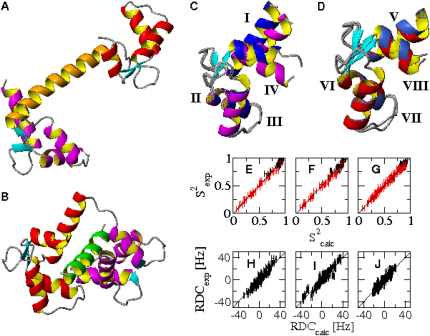
Structural and Dynamic Properties of the Ca^2+^-CaM and CaM-MLCK States (A and B) Ribbon diagram of the crystal structures of Ca^2+^-CaM (A) and CaM-MLCK (B). (C and D) Comparison between the solution structures of the two domains (NTD in magenta and CTD in red) of Ca^2+^-CaM and the corresponding ones in CaM-MLCK (blue). The alignment was optimized for residues 29–54 and 101–130 in NTD (C) and CTD (D), respectively. (E–G) Comparison of experimental and calculated S^2^ order parameters. Backbone S^2^ order parameters are shown in black, side-chain S^2^ order parameters in red. (E and F) NTD and CTD of Ca^2+^-CaM. (G) CaM-MLCK. Ensemble averages and standard deviations are shown. (H–J) Comparison of experimental and calculated RDCs. (H and I) NTD and CTD of Ca^2+^-CaM. (J) CaM-MLCK. Ensemble averages and standard deviations are shown.

Calmodulin (CaM) is a ubiquitous protein that plays a key role in calcium-mediated signal transduction. It has been shown that CaM binds and regulates more than 300 target proteins and that its structural plasticity is crucial for enabling its interaction with the diverse partners (Ikura and Ames, 2006). CaM consists of two homologous domains, the N-terminal domain (NTD) and the C-terminal domain (CTD), which are separated by an interdomain linker (Figures 1A–1D). Each domain is composed of two EF-hand helix-loop-helix motifs. These motifs occupy a “closed” conformation in the calcium-free (apo-CaM) state, in which the helices in the two pairs of EF hands are closely packed together. Ca^2+^ ligation during a calcium spike leads to an “open” state (Ca^2+^-CaM) in which significant changes in conformation in each EF-hand pair result in the exposure of a hydrophobic cleft in both domains (Zhang et al., 1995, Ikura, 1996). The exposure of this cleft increases the affinity of CaM for a wide range of binding partners (Osawa et al., 1998, Crivici and Ikura, 1995, Bayley et al., 1996).

The linker between the NTD and CTD also plays a key role in the function of CaM. Although this linker is seen as a long exposed helix in the crystal structure of Ca^2+^-CaM (Wilson and Brunger, 2000; Figure 1A), solution-state nuclear magnetic resonance (NMR) relaxation measurements (Barbato et al., 1992) have revealed that its central region is highly flexible in the Ca^2+^-CaM state. The importance of this flexibility in binding becomes apparent on examining the structures of Ca^2+^-CaM in complex with target peptides such as that derived from myosin light chain kinase (CaM-MLCK) (for a review, see Hoeflich and Ikura, 2002). In most structures of complexes, the NTD and CTD are effectively clamped together around a helical peptide (Figure 1B), although more recent structural studies have uncovered a variety of other binding modes (reviewed in Hoeflich and Ikura, 2002).

The mechanisms of conformational transitions that accompany the process of complex formation by proteins have been the subject of intense experimental and theoretical research for several decades (Monod et al., 1965, Koshland et al., 1966, Perutz et al., 1998, McCammon et al., 1976, Gerstein et al., 1994, Bui and McCammon, 2006). In recent years, a dynamic population shift model has been proposed according to which the structural transitions observed during complex formation can be described in terms of a variation in the equilibrium distributions of pre-existing populations that interchange dynamically in the absence of a binding partner (Kern and Zuiderweg, 2003, Boehr et al., 2006, Vendruscolo and Dobson, 2006, Faralado-Gómez and Roux, 2007, Arora and Brooks, 2007, Henzler-Wildman et al., 2007). In this model, consistent with the statistical view of protein folding (Bryngelson et al., 1995), a protein continuously samples a range of substates whose statistical weights are redistributed upon binding. In the case of CaM, NMR relaxation data indicate that in the absence of bound Ca^2+^, the CTD exists in a dynamic equilibrium between the closed apo state and an open conformation that is similar to that of Ca^2+^-CaM (Malmendal et al., 1999).

In this paper, we describe the determination of ensembles of structures representing the equilibrium fluctuations of the free and bound states of CaM. In this protocol, experimental NMR order parameters (S^2^) are used together with interproton distances derived from nuclear Overhauser effects (NOEs) as restraints in molecular dynamics simulations (Lindorff-Larsen et al., 2005, Richter et al., 2007). The method, as implemented here, allows extensive interdomain movements while reproducing accurately fluctuations on the picosecond to nanosecond timescale within the individual domains (see the Experimental Procedures). It combines the strength of NMR spectroscopy to provide measurements of structural and dynamic features at atomic level with the ability of molecular dynamics simulations to generate a wide range of conformations (Lindorff-Larsen et al., 2005, Richter et al., 2007). We apply this approach to determine the ensembles of structures representing the Ca^2+^-CaM and CaM-MLCK states and characterize the equilibrium population shifts in CaM upon binding.

Our results demonstrate that conformational features of the CaM-MLCK state are already present, although with a low statistical weight, in the ensemble of conformations representing the Ca^2+^-CaM state. We show that a population shift upon binding, which leads to much larger statistical weights for the bound-like conformations, is made possible through a specific organization of structural fluctuations that gives rise to correlated motions. We thus discuss a coupled equilibrium shift mechanism in which the ligand binds first to the CTD domain, and this event facilitates the further binding to the NTD domain.

## Results and Discussion

### Determination and Validation of Ca^2+^-CaM and CaM-MLCK Structural Ensembles

Simulated annealing cycles were used to generate multiple ensembles, each of 16 conformations, compatible with the experimental S^2^ values and representing the Ca^2+^-CaM and the CaM-MLCK states (Figures 1E–1G). The quality of these ensembles was assessed by using them to predict residual dipolar couplings (RDCs). The calculated ensemble-averaged Q-factors (see the Experimental Procedures) in the Ca^2+^-CaM state are 0.27 ± 0.1% and 0.30 ± 0.1% for the NTD and CTD, respectively (Figures 1H and 1I). This Q-factor for the NTD is actually better than that calculated from the high-resolution crystal structure (Wilson and Brunger, 2000) (0.40%), while that of the CTD is comparable (0.25%). The Q-factor calculated from the N-HN RDCs of the CaM-MLCK ensemble is 0.28 ± 0.1%, which is essentially the same as that for the crystal structure of the CaM-MLCK complex (0.27%) (Meador et al., 1992; Figure 1J).

**Figure 2:**
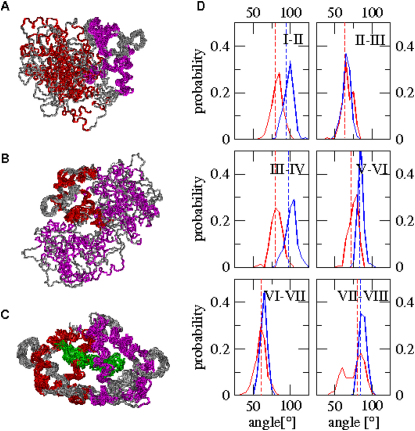
Structural Ensembles and Analysis of Their Intradomain Properties (A and B) Ensemble of structures (PDB code: 2K0E) representing the Ca^2+^-CaM state. Structures are aligned according to their NTD domains (magenta) and CTD domains (red), respectively. (C) Ensemble of structures (PDB code: 2K0F) representing the CaM-MLCK state. The ligand is shown in green. (D) Distribution of interhelical angles in the ensembles of Ca^2+^-CaM (red) and CaM-MLCK (blue), respectively. Interhelical angles in the RDC-refined solution structure of Ca^2+^-CaM and the X-ray structure of CaM-MLCK are indicated by dashed red and blue lines, respectively, and are shown to be close to the average values obtained through the calculations presented here.

To assess the heterogeneity of the structural ensembles, we determined the distribution of the root mean square (RMS) distances of the calculated ensembles from the RDC-refined structures of the NTD and CTD in Ca^2+^-CaM (Chou et al., 2001) and the X-ray structures of Ca^2+^-CaM and CaM-MLCK, respectively (Wilson and Brunger, 2000, Meador et al., 1992; see Figures S1A–S1F in the Supplemental Data available with this article online). The structures in the Ca^2+^-CaM ensembles have, on average, a large RMS deviation from the X-ray structure (Wilson and Brunger, 2000), 12.3 ± 3.1 Å (Figure S1A), as a consequence of the central linker connecting the NTD and CTD being highly flexible in solution. Indeed, since in our molecular dynamics simulations we restrain the motion within the molecular frame of the individual domains without interfering with their tumbling in solution, the calculated structures span a range of interdomain conformations that can deviate significantly from that observed in a crystalline environment (Figures 2A and 2B). By contrast, for the individual domains, the RMS deviation from the RDC-refined solution structures (Chou et al., 2001) is on average only 1.4 ± 0.3 and 1.8 ± 0.3 Å for the NTD and CTD, respectively (Figures S1B and S1C). In the case of the CaM-MLCK ensemble, despite the conformational heterogeneity enforced through the S^2^ restraints, the deviation of the entire complex from its crystalline counterpart (Meador et al., 1992) is on average only 1.5 ± 0.3 Å (Figure S1D). Therefore, through annealing cycles and ensemble-averaged simulations, NOEs and S^2^ restraints allow heterogeneous structural ensembles to be generated whose RMS deviations from the X-ray structure and the RDC-refined solution structures are very low.

These results indicate that the protocol used here provides structural ensembles that describe accurately the conformations presents in the Ca^2+^-CaM and the CaM-MLCK states. Moreover, we have also shown that the quality of the Ca^2+^-CaM ensemble is superior to that of ensembles generated with NOEs alone, or derived from classical molecular dynamics simulations or the superposition of the first 100 normal modes (see the Supplemental Data).

### Motions of the EF Hands in the Ca^2+^-CaM and CaM-MLCK States

Substantial variations exist in the orientations of the helical axes in the structures of the Ca^2+^-CaM ensembles. Interhelical angles, which are commonly used as indicators of structural changes between the open and closed states of CaM (Nelson and Chazin, 1998, Chou et al., 2001), fluctuate around their average orientations with amplitudes whose average and maximal values are 8° and 24°, respectively (Figure 2D); this latter value is consistent with estimates for the maximal amplitudes of helical motion of 20° (Chou et al., 2001). We observed slightly reduced average values (5°) for the fluctuations of the interhelical angles within the CaM-MLCK complex. In accord with the structural differences observed between the previously determined structures of Ca^2+^-CaM (Chou et al., 2001) and CaM-MLCK (Meador et al., 1992), the average interhelical angles between helices I-II, III-IV, and V-VI are significantly different in the free and the bound states.

The present study reveals that, despite the structural differences between the Ca^2+^-CaM and the CaM-MLCK states, interhelical angles are observed in the Ca^2+^-CaM state that correspond to those present in the most populated structures in the CaM-MLCK state, albeit with a low statistical weight. In the Ca^2+^-CaM ensemble there are few structures (less than 1%) in which the NTD interhelical angles (I-II and III-IV) are similar to those in the CaM-MLCK state (i.e., they deviate by less than 5° from the corresponding angles in the most populated structures of CaM-MLCK) (Table 1). By contrast, significantly more Ca^2+^-CaM structures (about 17%) have their EF-hands in the CTD as open as in the CaM-MLCK complex. These data indicate that structural fluctuations in the NTD and CTD of Ca^2+^-CaM allow the occasional sampling of conformations that are very similar to the predominant ones in CaM-MLCK, particularly in the CTD. However, the likelihood of the EF-hands in both domains being simultaneously in a complex-like conformation is lower than 1%.

**Table 1:** Percentage of Ca^2+^-CaM Structures with Properties Characteristic of the CaM-MLCK Ensemble

Property	NTD^a^	CTD^b^	NTD + CTD^c^
Interhelical angles (±5°)	0.4%	17.4%	0.1%
CαRMSD (±0.25 Å)	0.3%	2.5%	0.0%
Helical axes disposition (±10°)	0.5%	13.2%	0.3%
Methionine distances (±0.7 Å)	0.6%	8.5%	0.0%

a Property present in the NTD.

b Property present in the CTD.

c Property present in both domains concomitantly. The table provides the percentage of structures in the Ca^2+^-CaM ensemble with properties within the specified range from the predominant value of the CaM-MLCK ensemble.

### Intradomain Overlap between the Ca^2+^-CaM and CaM-MLCK States

We monitored the distributions of a range of other structural properties in the Ca^2+^-CaM and CaM-MLCK ensembles to verify in further detail their intradomain overlap.

**Figure 3:**
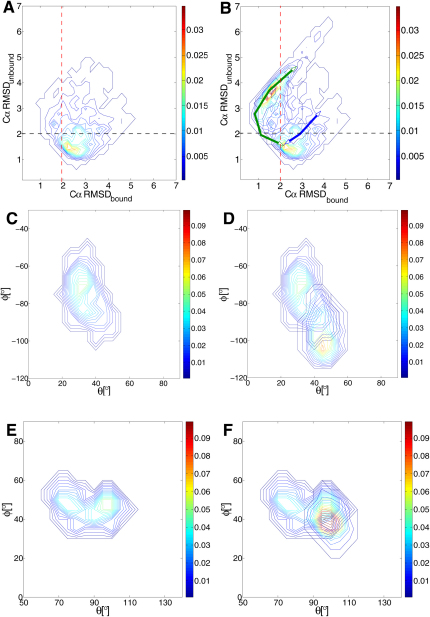
Comparison of Structural Properties in the Ca^2+^-CaM and CaM-MLCK Ensembles After superimposing the members of both ensembles on the crystal structure of CaM-MLCK as described in the Supplemental Data, properties of the nonaligned helices were determined and compared. (A) Probability distribution of the RMSD of helices I and IV with respect to the RDC-refined solution structure of Ca^2+^-CaM (CαRMSD_unbound_) and the crystal structure of CaM-MLCK (CαRMSD_bound_) in the Ca^2+^-CaM ensemble. (B) The probability distribution of the RMSD in the CaM-MLCK ensemble is added to the probability distribution shown in A. Projection of the third (green) and fourth (blue) modes of motion in the NTD on CαRMSD_unbound_ and CαRMSD_bound_, respectively. (C and E) Probability distributions of the positions of helix I and helix VIII, respectively, in the Ca^2+^-CaM ensemble. (D and F) Probability distributions of the positions of helix I and helix VIII in CaM-MLCK ensembles are added to the ones shown in C and E, respectively.

We performed a rigid body alignment of helices II and III in the NTD with respect to the RDC-refined solution structure of Ca^2+^-CaM as well as the crystal structure of CaM-MLCK to accentuate the differences between the Ca^2+^-CaM and CaM-MLCK ensembles (see the Supplemental Data). In Figures 3A and 3B, we show values of the Cα RMSDs of the nonaligned helices I and IV from their counterparts in the solution structure of Ca^2+^-CaM and the crystal structure of CaM-MLCK. Analysis of the Ca^2+^-CaM ensemble (Figure 3A) indicates that 56% of the conformations have helices I and IV within 2 Å of their positions in the solution structure (conformations below the black dashed line). In the remaining structures of the ensemble, however, helices I and IV have a significantly higher Cα RMSD_unbound_, reaching values of up to 5 Å. Importantly, in some of the conformations that have high values of Cα RMSD_unbound_, helices I and IV are found to deviate by less than 2 Å from the structure they adopt in the complex (left of the red dashed line). This region of the projection (above the black dashed line and left of the red dashed line) also accommodates the structures most populated in the bound state (Figure 3B). Consequently, about 0.3% of the NTD conformations in the Ca^2+^-CaM ensemble correspond to those of highest probability in the CaM-MLCK ensemble (Table 1). The analysis for the CTD shows similar results for helices V and VIII when helices VI and VII are aligned (Figure S5). In the case of the CTD, however, the structural differences between the two ensembles are significantly smaller, and there is an almost 10-fold increased probability of finding structures in the Ca^2+^-CaM ensemble with low Cα RMSD relative to the complex.

**Figure 4:**
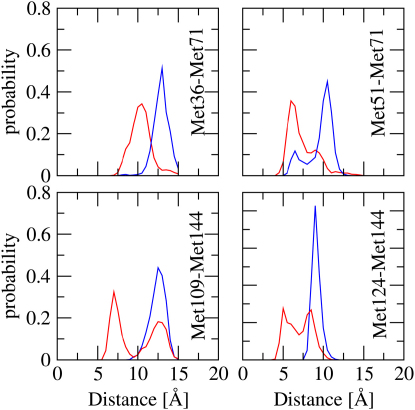
Distribution of Methionine Distances in the Ensembles of Ca^2+^-CaM, Red, and CaM-MLCK, Blue

We also monitored the spatial orientation of the helical axes (Figures 3C–3F), the distributions of intermethionine distances (Figure 4), and the tertiary structure of each EF-hand (Figure S6) to compare the structural characteristics of the ensembles that we determined. Consistent with the analysis described above, a few conformations of the Ca^2+^-CaM ensemble have structural properties that are close to the ones found for the majority of structures in the CaM-MLCK ensemble.

### Correlated Intradomain Motions in the Ca^2+^-CaM State Leading Toward the CaM-MLCK State

**Figure 5:**
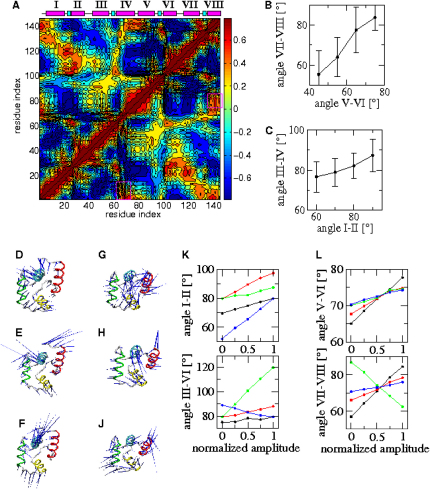
Analysis of the Backbone Motions in Ca^2+^-CaM (A) Correlated backbone motions. The position of the helices and the Ca^2+^ binding sites are indicated by magenta and cyan bars, respectively. (B and C) Correlation in interhelical angles (ρ_CTD_ = 0.65 and ρ_NTD_ = 0.15) (*P* < 0.0001). Averages and standard deviations of binned angles are shown. (D–J) Porcupine plots of the first three modes in NTD (D–F) and CTD (G–J). (K and L) Effect of the first (black), second (red), third (green), and fourth (blue) mode on the interhelical angles in NTD (K) and CTD (L). Motions were started from conformations with “closed” interhelical angles I-II and V-VI, respectively.

To obtain insight into the type of motions that bring Ca^2+^-CaM close to the CaM-MLCK state, we performed a detailed analysis of the dynamics of the Ca^2+^-CaM state. The extent to which backbone motions are correlated in the Ca^2+^-CaM ensemble is illustrated in Figure 5, and the cross-correlation matrix (Figure 5A) clearly indicates that the changes in backbone structure are achieved by correlated rigid body motions. Helices I and IV in the NTD, as well as V and VIII in the CTD, move in a concerted way. By contrast, helices I(V) and IV(VIII) exhibit anticorrelated motions with helices II(VI) and III(VII), respectively, a finding that is consistent with their involvement in hinge or scissor-like motions. The correlation in rigid body backbone motions, which is more pronounced in the CTD, result in a concerted “opening” and “closing” of the EF-hands in the two domains (Figures 5B and 5C).

Whereas the cross-correlation analysis highlights regions of the structures that move in a concerted manner, the eigenvectors and eigenvalues of a principal component analysis (PCA) emphasize the direction and amplitude of the dominant modes of the motions in a protein. The PCA of the NTD and CTD in the Ca^2+^-CaM ensemble (Figures 5D–5J) suggests that the dynamics in the two domains are governed by complex hinge motions of the helices that are more than simple scissor-like motions. However, an opening and closing of the interhelical angles (Figures 5K and 5L) is common to the first four modes, which contribute up to 75% of the overall intradomain motions (Figure S7). In the NTD, the first three modes of motions lead to a simultaneous opening or closing of the interhelical angles I-II and III-IV; the amplitude of opening is, however, most significant in the third mode. The projection of this mode on the progress variables CαRMSD_bound_ and Cα RMSD_unbound_ (Figure 3B) shows that it induces sampling of complex-like NTD conformations in the Ca^2+^-CaM state. This type of projection indicates how much a particular type of motion of the protein (in this case a specific mode) moves the protein toward a particular direction (e.g., along a reaction coordinate). The fourth mode in the NTD, by contrast, opens one EF-hand while closing the other one (Figure 5K, blue line), and does not lead to the sampling of complex-like NTD conformations (Figure 3B). In the CTD, the first mode has the most pronounced effect on the interhelical angles, opening (closing) simultaneously both EF-hands. This mode is principally responsible for the sampling of complex-like conformations in the CTD of the Ca^2+^-CaM state (Figure S5C), while the remaining modes have sequentially less impact on the interhelical angles.

The detailed study of the intradomain dynamics therefore indicates the presence of complex hinge motions of the helices. However, the opening movements of the two EF hands in each domain are clearly coupled, an effect that increases the likelihood of sampling conformations resembling those present in the CaM-MLCK state. Indeed, the PCA suggests that motions with the greatest amplitude present in both domains are those that generate complex-like substates. The correlation in intradomain motions is, nevertheless, not equally pronounced in both domains. In the CTD, the coupling between the two EF hands is significantly stronger than in the NTD, and again it is the first motion mode that is the one responsible for the sampling of complex-like substates.

The analysis of various structural indicators and intradomain motions reveals that conformations are sampled in the Ca^2+^-CaM state, which have intradomain properties corresponding to the predominant ones in the CaM-MLCK state. This finding suggests that binding of the ligand may occur by an equilibrium shift mechanism. However, as stated by Yu and Koshland (Yu and Koshland, 2001), the presence of ligand-competent conformations does not inevitably mean that the ligand binds first to these structures. If the sampling of complex-like structures or the binding process are too slow the equilibrium shift “pathway” may not be the favored one. The transition between different substates in Ca^2+^-CaM is very fast (nanoseconds), and MLCK associates with Ca^2+^-CaM at a rate of about 10^8^ M^−1^ s^−1^ (Kasturi et al., 1993); we can assume, therefore, that MLCK interacts first with the small population of molecules in the Ca^2+^-CaM ensemble that is complex-like. However, the concomitant presence of complex-like structural features in both domains is rare. Our analysis shows that the CTD is more finely tuned to sample conformations with complex-like properties. Because of the higher likelihood of sampling complex-like conformations in the CTD, we suggest that the initial binding and shift in populations will occur in this domain. This conclusion is consistent with biophysical data that indicate that the interaction of CaM with MLCK proceeds via an intermediate complex in which the CTD of CaM interacts first with MLCK (Bayley et al., 1996).

### Characterization of the Interdomain Motions

**Figure 6:**
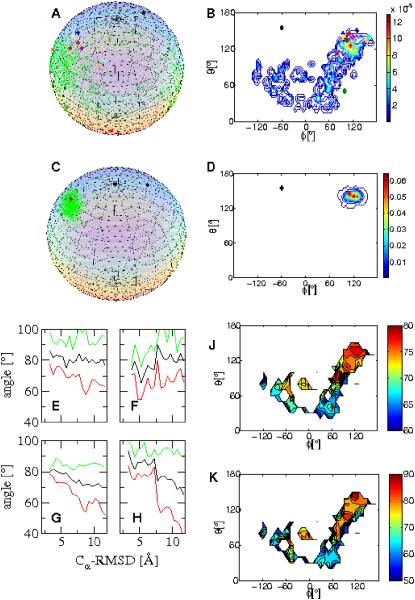
Analysis of the Interdomain Motions and of Their Coupling to Intradomain Structural Changes (A) Position of the vector representing the axis of helix IV and V in the Ca^2+^-CaM ensemble on a unit sphere. The Cα atom of residue 79 (in the middle of the flexible linker of CaM) is placed at the center of the sphere. The position of helix V, on which structures were aligned (i.e., fixed), is shown as a black diamond, and that of helix IV in the crystal structure of Ca^2+^-CaM and the crystal structure of CaM-MLCK are indicated by a red square and diamond, respectively. The positions of helix IV in the Ca^2+^-CaM ensemble are indicated by green dots; those found in different CaM complexes are indicated by additional diamonds (fully Ca^2+^ loaded) and crosses (partially loaded) (for details, see Figure S8). (B) Distribution of the axis of helix IV in the Ca^2+^-CaM ensemble in polar coordinates. The positions found in the structures of CaM complexes are indicated as in (A). (C) Position of the vector representing the axis of helix IV and V in the CaM-MLCK ensemble on a unit sphere. (D) Distribution of the axis of helix IV in the CaM-MLCK ensemble in polar coordinates. (E–H) Interhelical angles in the Ca^2+^-CaM ensemble as a function of the CαRMSD from the crystal structure of CaM-MLCK. The average, smallest, and largest angles of each bin are shown in black, red, and green, respectively. Interhelical angles I-II, III-IV, V-VI, and VII-VIII are shown in (E), (F), (G), and (H), respectively. (J and K) Interhelical angles V-VI and VII-VIII, respectively, as a function of the interdomain position of helix IV in the Ca^2+^-CaM ensemble. The position of the helix is calculated as in (B).

**Figure 7:**
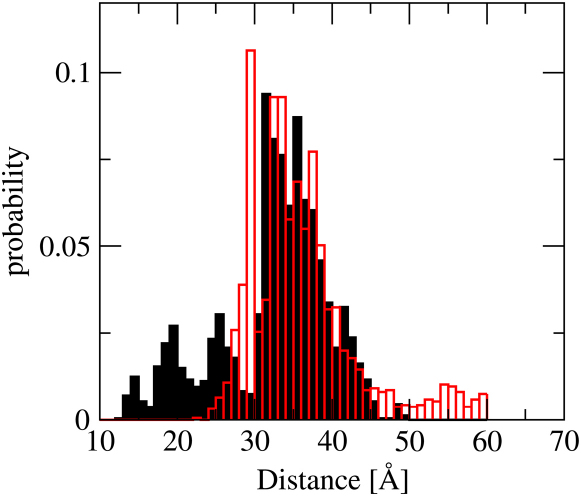
Comparison between FRET-Derived Distances and the Corresponding Ones Calculated from the Ca^2+^-CaM Ensemble The distance distribution measured by FRET between a donor fluorophore and acceptor fluorophore on residues 34 in the NTD and 110 in the CTD of Ca^2+^-CaM is shown in red; the distance distribution calculated from the Ca^2+^-CaM ensemble is shown in black. See Johnson, 2006.

Because the method introduced in this study restrains the motions within individual domains without affecting their overall tumbling behavior, the NTD and the CTD of the Ca^2+^-CaM state sample a wide range of interdomain conformations (Figures 2A and 2B). We therefore calculated a generalized order parameter (S^2^global) for the interdomain motion (see the Supplemental Data). For the Ca^2+^-CaM ensemble an S^2^global value of 0.13 ± 0.03 was obtained, which is only slightly higher than that derived recently from experimental data (0.02) (Bertini et al., 2004). In the CaM-MLCK ensemble, by contrast, the interdomain motions are far more restricted, a situation that is reflected in the very high value obtained for the order parameter (S^2^global = 0.89 ± 0.02). To investigate the global orientations of the two CaM domains, we monitored the distribution of the position of helix IV while keeping helix V fixed (Figures 6A–6D). Interestingly, helices IV and V frequently adopt orientations in the structures of the Ca^2+^-CaM ensemble that are close to their orientations in the complexed state. Moreover, the distances between residues 34 in the NTD and 110 in the CTD, calculated from the structures of the Ca^2+^-CaM state that we determined here, are comparable to those measured by FRET experiments (Johnson, 2006; Figure 7). A large majority of the distances between the donor and the acceptor ranges in both cases between 30 and 40 Å. Experimentally, the time scale for interdomain reorientation and formation of compact conformations has been estimated to be about 3 ns (Baber et al., 2001). Moreover, it has also been suggested that largely open interdomain conformations can be sampled on the millisecond timescale (Bertini et al., 2004). Despite the fact that the interdomain motions were not restrained in the simulations, it appears from these results that interdomain dispositions similar to those observed for the complex are preferred by the intrinsic steric properties of the protein.

### CaM Explores a Variety of Complex-like Interdomain Conformations in the Ca^2+^-CaM State

**Figure 8:**
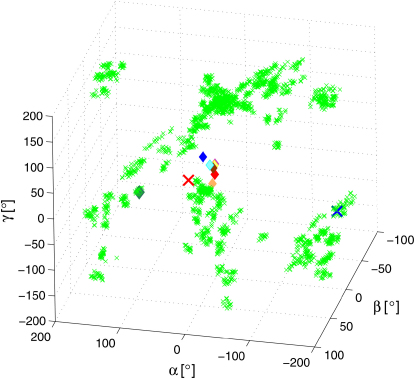
Interdomain Motions in the Ca^2+^-CaM Ensemble Shown are the Euler angles defining the orientation of the NTD with respect to the CTD. The angles calculated for the Ca^2+^-CaM ensemble are shown as green crosses, those found in complexes of CaM are indicated as in Figure 6.

We examined the extent to which the conformational space accessible within the Ca^2+^-CaM state includes the conformations found in the X-ray structures of CaM when in complex with a variety of peptides and proteins. We therefore analyzed the relative orientations of the axes of helices IV and V (Figures 6A–6D) and calculated the Euler angles defining the orientations of the NTD with respect to CTD (Figure 8) in Ca^2+^-CaM and representative structures of all complexes of CaM with peptides/proteins that have been determined so far (see the Supplemental Data). Figures 6A–6D and Figure 8 indicate that the conformational space sampled in the Ca^2+^-CaM state covers essentially all the global orientations found so far in complexes of CaM. Moreover, the sterically preferred interdomain disposition adopted by Ca^2+^-CaM deviates by only a few degrees from that of the conformations found in the majority of the complexes formed by CaM. However, the probability of sampling substates with this interdomain topology is at least 10 times higher in the presence of the ligand than in its absence. In most but not all complexes, the NTD and CTD clamp around a target peptide; in particular, the global conformations of CaM in complex with the K+ channel (Schumacher et al., 2001) and the anthrax edema factor (Shen et al., 2002) are significantly different from the majority. In these cases, only one of the two domains of CaM is ligated to Ca^2+^ and, instead of wrapping around the peptide molecule, the two domains bind to the target proteins in a far more open interdomain conformation; this conformation is present in the Ca^2+^-CaM ensemble, but only at a very low statistical weight (Figures 6A and 6B).

### Coupling between Intradomain and Interdomain Motions

To establish whether intradomain and interdomain properties of CaM change in a concomitant manner within the Ca^2+^-CaM ensemble, we analyzed the changes in the interhelical angles as a function of a range of parameters describing the interdomain organization of the NTD and CTD.

First, the interhelical angles I-II, III-IV, V-VI, and VII -VIII were projected as a function of the average RMS deviations of CaM from the crystal structure of CaM-MLCK (Figures 6E–6H). We observed a clear trend for the interhelical angles V-VI and VII-VIII to be more open in conformations with a topology similar to that adopted by CaM upon binding MLCK. In structures with a RMS deviation from the crystal structure of CaM-MLCK lower than 3.5 Å, the interhelical angles V-VI and VII-VIII are 80 ± 2° and 86 ± 5°, respectively, which are close to those in the crystal structure of CaM-MLCK (81° and 82°, respectively). Those structures of the Ca^2+^-CaM ensemble that have only small deviations in their interdomain angle from that observed in CaM-MLCK are also found to have the smallest deviations in their interhelical angles V-VI and VII-VIII (Figures S9C and S9D). In addition, we monitored the interhelical angles as a function of the orientation of the axis of helix IV (while keeping helix V fixed) in the various structures and observed that the interhelical angles V-VI and VII-VIII are higher when complex-like global conformations are sampled (Figures 6J and 6K, compared with Figures 6B and 6D). By contrast, intradomain and interdomain motions appear to be less strongly linked in the NTD. The trend toward larger values within compact conformations is modest for the interhelical angle I-II, and absent for helices III and IV. In particular, a large interhelical angle III-IV (>85°), which is necessary for binding, is usually found in conformations that have a large RMS deviation from the crystal structure of CaM-MLCK (Figure 6F).

These results suggest that a correlation is present, at least in the CTD, between the occasional adoption, in the absence of a ligand, of intradomain and interdomain conformational properties characteristic of the CaM-MLCK state. However, the coupling between interdomain and intradomain motions is not very tight, with large interhelical angles observable in both domains within conformations of the Ca^2+^-CaM ensemble that are not compact and that have a significant RMS deviation from the crystal structure of CaM-MLCK (green line Figures 6E–6H).

### Interdomain Coupling of Intradomain Motions

Finally, we investigated how intradomain motions are coupled between the domains. The cross-correlation matrix shown in Figure 5A clearly indicates that concerted motions occur also across domains, not just within each domain. Helices I, IV, V, and VII exhibit correlated motions, but they are predominantly anticorrelated with the motions of the other helices. In particular, motions in the interdomain region, including helices IV and V as well as the short interdomain linker, are strongly coupled. As both of these helices are linked in their motions with the other helices within the respective domains (see above), the interdomain regions appears to play a key role in the communication between the NTD and CTD.

A large body of experimental evidence on apo- and ligand-bound CaM suggests a structural coupling between the two domains (Martin et al., 1992, Sorensen and Shea, 1998, Yazawa et al., 1992). Although it has not been possible to determine precisely whether, and to what extent, initial binding of a target protein or peptide by the CTD enhances the affinity for binding by the NTD (Crothers and Metzger, 1972), the linked thermodynamics of Ca^2+^ and ligand binding makes cooperative target binding highly likely. It appears, therefore, that altering the distribution of structural properties in one domain upon binding is capable of affecting those in the other domain. To test this possibility, the structure for Ca^2+^-CaM in complex with the N-terminal portion of the CaM-binding domain of the plasma membrane calcium pump, C20W (Elshorst et al., 1999), was analyzed. In this case, the CTD of Ca^2+^-CaM binds to C20W with no detectable contacts between C20W and the NTD. As expected, the interhelical angles in the CTD are consistent with those of a bound structure (angles V-VI and VII-VII are 87 ± 3° and 104 ± 5°, respectively). Interestingly, the interhelical angles, I-II and III-IV, in the NTD are also very large (112 ± 6° and 98 ± 6°, respectively). Indeed, these latter values are among the largest found in any structures of CaM in complex with a protein or peptide, despite the fact that the NTD is not detectably involved in the binding with C20W.

The structural correlations that we observed between the two domains in the Ca^2+^-CaM ensemble provide a structural basis for a variety of experimental observations that indicate interdomain communication in CaM. The coupling between the two domains is of statistical nature and is strongly dependent on the asymmetry in the structure and dynamics of the NTD and CTD.

### Sequential Population Shift upon Substrate Binding

Our results show that Ca^2+^-CaM is in dynamic equilibrium with a series of complex-like states. Because there is fast exchange between substates and a high association rate, one may assume that ligand binding occurs by an equilibrium shift mechanism. Hence, the substrates may in principle bind to Ca^2+^-CaM conformations that are simultaneously complex-like in both domains. However, this type of conformation is sampled very rarely, because Ca^2+^-CaM is not completely symmetric and the CTD is more finely tuned than the NTD to adopt conformations resembling those normally populated by CaM-MLCK. Therefore, our analysis shows in structural terms why MLCK binding is far more likely to take place at the CTD prior to binding to the NTD. These results are strongly supported by the available biophysical evidence that shows that the “wrap-around” interactions are initiated by the binding of the CTD (Liu et al., 2006).

We have shown that structural changes are not only correlated within the two domains of Ca^2+^-CaM but also between them. Consequently, the population shift induced by initial target binding at the CTD will alter the equilibrium population of unbound conformations within the NTD, such that new substates become accessible and complex-like conformations are more frequently populated. Thus, the initial binding of the ligand by the CTD changes the free energy landscape of the NTD in such a way that substates necessary for ligand binding by the NTD are more readily accessible.

### Versatility and Efficiency in Ligand Binding

The intradomain and interdomain structural variability of CaM provides the basis for the coupling between Ca^2+^ binding and exposure of hydrophobic residues, both essential factors determining the ability of CaM to bind to its targets. This type of versatility is made possible through the presence of a flexible linker connecting the two domains as well as a structural and dynamical asymmetry within and between these two domains. The approach adopted in this study has enabled us to characterize in great detail both the structural and dynamical differences between the two domains and to reveal that large amplitude modes of motion have different structural effects in the NTD and CTD and create a high versatility and specificity for binding to a variety of substrates.

Creating versatility by introducing asymmetry in duplicated motifs may, in principle, come at the expense of a reduced efficiency to bind a specific ligand. In the case of CaM, however, we have been able to show that the principal dynamic modes in the Ca^2+^-CaM state are essential to direct efficiently the sampling of the conformational space toward complex-like substates. In the CTD, it is the first mode that leads to CaM-MLCK-like substates, whereas the third mode has the same effect in the NTD. Overall, our data indicate that the design of this protein makes it possible to achieve structural variability without reducing significantly the efficiency in binding target proteins.

### Coupled Equilibrium Shifts on a Dynamic Energy Landscape

**Figure 9:**
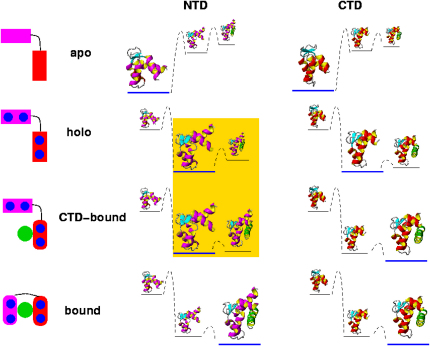
Free Energy Landscape of CaM Illustrating the Coupled Equilibrium Shift Mechanism Based on our findings and previous experimental data (Malmendal et al., 1999), we suggest that the different states of CaM are in a dynamic equilibrium and, in particular, that shifts in the equilibrium are coupled between the two domains. The binding of Ca^2+^ ions initially shifts the equilibrium from the apo to the holo state (Ca^2+^-CaM). Binding of the MLCK ligand takes place first at the CTD, which assumes the bound conformation. This event increases the likelihood of sampling high-affinity substates in NTD (highlighted in yellow), which eventually leads to binding and an equilibrium shift in this domain.

Our results suggest that CaM binds to different targets through a mechanism of “coupled equilibrium shifts” (Figure 9). Preferential prebinding of a target to a high-affinity substate of one of the domains—the CTD in the case of MLCK—shifts the equilibrium population in this domain and promotes the progression of the binding reaction along a particular channel on the free energy landscape. Other targets that bind different substates of Ca^2+^-CaM will promote progressions along different channels on this landscape. In this view, initial binding to one domain induces a shift in the free energy landscape, resulting in an increased population of substates in the other domain that have a high affinity for the ligand. Therefore, the binding to one domain effectively facilitates the subsequent binding to the other domain. This view is consistent with a previously described model of CaM binding (Bayley et al., 1996, Liu et al., 2006, Slaughter et al., 2007), considering it within the concept of a dynamic energy landscape of a protein (Faralado-Gómez and Roux, 2007, Arora and Brooks, 2007, Henzler-Wildman et al., 2007) and related to mechanisms of binding and conformational change that have been proposed for other proteins (Boehr et al., 2006). The mechanism of coupled equilibrium shifts allows rationalizing the remarkable characteristic of many proteins to combine high versatility with high efficiency.

## Experimental Procedures

### Structure Calculations

The atomic coordinates of the of the RDC-refined solution structure of Ca^2+^-CaM (PDB codes: 1J7O, 1J7P) (Chou et al., 2001) and the crystal of the CaM-MLCK complex (PDB code: 1CDL) (Meador et al., 1992) were used as starting structures for the molecular dynamics calculations. The calculations were performed using CHARMM (Brooks et al., 1983) with the CHARMM22 force-field. The structures were solvated in an 8 Å shell of TIP3 water molecules. A soft boundary potential was used to prevent water molecules from escaping (Beglov and Roux, 1994).

All calculations used an atom-based truncation scheme with a list cutoff of 14 Å, a nonbond cutoff of 12 Å, and the Lennard-Jones smoothing function initiated at 10 Å. Electrostatic and Lennard-Jones interactions were force switched. MD simulations used a 2 fs integration time step and SHAKE for covalent bonds involving hydrogen atoms (Ryckaert et al., 1977).

As discussed previously (Bonvin and Brunger, 1996, Bürgi, 2001), it is difficult to demonstrate that the use of ensemble-averaged restraints provides the correct statistical weights of the conformations in the resulting ensembles. However, in a previous study, we have shown that use of the MUMO method enables the accurate recovery of all the pairwise distance distributions in a test case when such distributions are known exactly, and also provides very low Q factors for RDCs when experimental NOEs and S^2^ data are used as restraints (Richter et al., 2007). These results indicate that the statistical weights are effectively calculated with good accuracy when the MUMO procedure is used.

In restrained ensemble-averaged simulations, structural information from NOEs is combined with NMR relaxation data in the form of order parameters (S^2^) using an energy function

(1) $$E_{\mathrm{tot}}=E_{\mathrm{CHARMM}}+E_{\mathrm{NOE}}+E_{S^{2}}$$

in which $E_{\mathrm{CHARMM}}$ is the CHARMM22 force field (Brooks et al., 1983) and $E_{\mathrm{NOE}}$ and $E_{S^{2}}$ are the energies associated with the NOEs, and S2 ensemble-averaged restraints, respectively. The structure calculations consisted of 10 cycles of simulated annealing. After each cycle, structures were equilibrated at 300K and used for further analysis (for more details, see the Supplemental Data). The resulting structures are deposited in the PDB (PDB codes: 2K0E and 2K0F).

Residual dipolar couplings were back-calculated from the structural ensembles using singular value decomposition to fit the alignment tensor. The RDCs deposited with the PDB codes 1J7O and 1J7P (Chou et al., 2001) were used for fitting the alignment tensors of NTD and CTD, respectively, in the Ca^2+^-CaM ensembles. N-HN RDCs of the CaM-MLCK were used to fit the alignment tensors of the CaM-MLCK ensembles (provided by M. Ikura). The quality of agreement with experimental RDC data was assessed by calculating the Q-factor:

(2) $$Q=\frac{\sqrt{\sum {\left({\mathrm{RDC}}_{\mathrm{calc}}-{\mathrm{RDC}}_{\exp}\right)}^{2}}}{\sqrt{\sum {\left({\mathrm{RDC}}_{\exp}\right)}^{2}}}$$

The cross-correlation coefficient $C_{\mathrm{ij}}$ between the displacement of Cα atoms *i* and *j* was calculated as

(3) Cij=〈ΔriΔrj〉/(〈Δri2〉〈Δrj2〉)12

where $\Delta r_{i}$ is the displacement from the average position of the Cα atom i. To analyze the flexibility of the two domains in the Ca2+-CaM ensembles, we used the PCA for structural ensembles proposed by Nilges and coworkers (Abseher et al., 1998; see the Supplemental Data).

## Accession Numbers

Structures have been deposited in the PDB under codes 2K0E and 2K0F.

## Supplemental Data

Document S1. Nine Figures, Two Tables, Supplemental Results, Supplemental Experimental Procedures, and Supplemental References
