# Supplementary material for: A Coupled Equilibrium Shift Mechanism in Calmodulin-Mediated Signal Transduction
Source: Structure. 2008 May 7;16(5):736–46. doi: 10.1016/j.str.2008.02.017 (PMC2428103; doi:10.1016/j.str.2008.02.017)
Supplement: Document S1. Nine Figures, Two Tables, Supplemental Results, Supplemental Experimental Procedures, and Supplemental References [file mmc1.pdf]

Structure *16*

Supplemental Data

A Coupled Equilibrium Shift Mechanism in Calmodulin-Mediated Signal Transduction

Jörg Gsponer, John Christodoulou, Andrea Cavalli, Jennifer M. Bui, Barbara Richter, Christopher M. Dobson, and Michele Vendruscolo

Supplemental Results

Comparison with Alternative Simulations Techniques

In order to assess the effect of the use of NOEs and S^2 order parameters as restraints in the molecular dynamics simulations that we present here, we compared properties of the Ca^{2+}-CaM ensemble to those of four control ensembles that we determined with alternative simulation techniques.

Simulations with NOE Restraints. To generate the first control ensemble, we carried out restrained molecular dynamics simulations using only NOE data. In order to test whether the broad fluctuations observed in Ca^{2+}-CaM ensemble can be reproduced by using only NOE data, within the approach that we described here, a weak force constant α_{NOE} was applied during the annealing cycles (see Supplemental Experimental Procedures). The resulting backbone S^2 order parameters are lower than the experimental ones (by 0.07 in the NTD and by 0.12 in the CTD, Figures S2A and S2B and Table S1). In addition, Q-factors of 0.36 and 0.39 for the NTD and CTD, respectively, indicate a lower structural quality compared to the Ca^{2+}-CaM ensemble determined by using NOEs and S^2 order parameters as restraints in the simulations. Thus, although by lowering the α_{NOE} force constant it is possible to generate conformations with significant structural heterogeneity, finding a value for the α_{NOE} force constant that reproduces the experimentally observed heterogeneity accurately has not been possible because lowering the α_{NOE} force constant increases the occurrence of NOE violations in the determined structures and, therefore, introduces an uncertainty on the quality of the average structure (Q-factors).

Normal Mode Analysis. We generated a second control ensemble by performing a normal mode analysis of Ca^{2+}-CaM and superimposing the first 100 modes. In this case, we found values of the S^2 order parameters that were higher than the experimental ones (by 0.05 in the NTD and by 0.12 in the CTD, Figures S2A and S2B and Table S1). In addition, Q-factors of 0.39 and 0.6 for the

NTD and CTD, respectively, show that this ensemble does not accurately reproduce the Ca^{2+}-CaM state. Hence, we have been able to reproduce the dynamics of CaM occurring on the picosecond to nanosecond time scale only with a rather low accuracy by using this type of approach. In agreement with these results, it has been reported previously that anhormonic motions are crucial for the explanation of NMR order parameters (Best et al.).

Unrestrained Molecular Dynamics Simulations. Standard (i.e. unrestrained) molecular dynamics simulations were used to generate two additional control ensembles. In the first simulation, CaM was solvated in a water shell with the same setup as in the restrained molecular dymamics simulations. In the second simulation, CaM was solvated in a orthorhombic box. Both simulations were carried out at 300 K for 2 ns (see Supplemental Experimental Procedures). From both trajectories, 200 conformations were extracted and their structural properties analysed. We found that the backbone S^2 order parameters are lower than the experimental ones in the structural ensembles extracted from both these control simulations (Figures S2C and S2D and Table S1). In addition, the ensemble averaged Q-factors for the NTD and CTD are 0.43 and 0.61, respectively, for the ensemble extracted from the water-shell simulation and 0.42 and 0.55, respectively, for the ensemble extracted from the simulation of Ca^{2+}-CaM in a water box. To assess the quality of these structural ensembles further, we calculated the backbone RMSD of their members with respect to the RDC-refined structures of Ca^{2+}-CaM (Chou et al.) (Figure S3). In both unrestrained simulations, the backbone RMSDs from the RDC-refined solution structures are significantly higher than the ones calculated for the structures generated by the molecular dynamics simulations with S^2 and NOE experimental restraints. It is, however, possible that by carrying out longer simulations or using other force fields, e.g. with a more sophisticated treatment of the electrostatics, structural ensemble can be generated in a better agreement with NMR data.

Structural Alignments Performed to Analyse the Overlap in Intradomain Properties between the Ca^{2+}-CaM and CaM-MLCK Structural Ensembles

The structural differences between the members of the Ca^{2+}-CaM and CaM-MLCK ensembles are emphasised when the structures are aligned to optimise the overlap of the structural elements that are more similar in the two ensembles. Figure 2D shows that the angles between helices II and III in the NTD (residues 29-54) and VI and VII in the CTD (residues 101-130) are almost identical in the two ensembles. The pairwise RMS deviation of residues that define these helical regions, 29-54 and 101-130, respectively, calculated between all members of the two ensembles is on average below 1.0 Å. In addition, root mean square distance differences, RMSDDs (Nelson and Chazin)

(see Supplemental Experimental Procedures), calculated between all structures of the Ca^{2+}-CaM and CaM-MLCK ensembles indicate that the structural differences between the Ca^{2+}-CaM and CaM-MLCK states are the lowest for residues 29-54 and 101-130, respectively (Table S2). In addition, the distribution of the RMSDDs shown in Figure S4 clearly shows that the structural differences between Ca^{2+}-CaM and CaM-MLCK are much more pronounced in the other parts of the structure of CaM. Therefore, structures were first overlaid on residues 29-54 and 101-130, respectively, and then the properties of the non-aligned regions analysed.

Supplemental Experimental Procedures

Detailed Description of the Restrained Ensemble-Averaged Molecular Dynamics Protocol

Restrained molecular dynamics techniques were used in combination with ensemble simulations to obtain structural ensembles that satisfy NOE and S^2 restraints. The use of the S^2 order parameters as restraints enables the structural heterogeneity of the native state ensemble to be sampled (Best and Vendruscolo). We have carried out repeated cycles of simulated annealing (Lindorff-Larsen et al.) (see below) in order to increase the efficiency of the sampling. In addition, while NOE distances and S^2 order parameters were initially enforced across sixteen replicas to get structural ensembles (Lindorff-Larsen et al.), a recently established protocol (MUMO) where S2 order parameters are enforced across sixteen replicas but NOE distances, on the other hand, averaged across only two replicas improved the quality of the calculated ensembles (for more details see (Richter et al.)). We used this modified protocol (MUMO) to determine the ensembles of structures representing the Ca^{2+}-CaM and CaM-MLCK states, respectively.
The restraint energy was implemented as (Best and Vendruscolo)

$$E_X(\rho,t) = \begin{cases} \frac{\alpha_X}{2}(\rho-\rho_0)^2 & \text{if } \rho(t) > \rho_0(t) \\ 0 & \text{if } \rho(t) \le \rho_0(t) \end{cases}, \qquad [1]$$

where X corresponds either to NOE, or S^2, α_X is the force constant associated with each type of restraint, and $\rho_0(t)$ is defined as (Paci et al.)

$$\rho_0(t) = \min_{0\le\tau\le t} \rho(\tau) \qquad [2]$$

In case of the NOE restraints

$$\rho_{NOE}(t) = \frac{1}{N_{NOE}} \sum \left(d_{NOE}^{\exp} - d_{NOE}^{ens} \right)^2 \qquad \textbf{[3]}$$

where the sum is taken over the number N_{NOE} of experimental NOE distances, and

$$d_{NOE}^{ens} = \left(\frac{1}{N_{rep}} \sum^{N_{rep}} d_{NOE}^{struct^{-3}} \right)^{-1/3} \qquad \textbf{[4]}$$

where N_{rep} is the number of molecules (replicas) used in the ensemble-averaged simulations. The d_{NOE}^{ens} distances were allowed to vary freely between their experimental upper and lower bounds. In order to compute the distances d_{NOE}^{struct} within individual molecules we considered all atom pairs associated with a given NOE restraint

$$d_{NOE}^{struct} = \left(\sum r_{ij}^{-6} \right)^{-1/6} \qquad \textbf{[5]}$$

where the sum is taken over all equivalent atoms. For Ca^{2+}-CaM, NOE-derived distance restraints were obtained from the PDB (Berman et al.) (Finn et al.) (Bentrop et al.). Distances incompatible with the RDC-refined solution structures of the NTD and CTD [1] (violation > 1 Å) were removed. To define a starting interdomain orientation of the two domains of Ca^{2+}-CaM, the RDC-refined solution structures of NTD and CTD were aligned with the corresponding domains in the crystal structure of Ca^{2+}-CaM (PDB 1EXR) (Wilson and Brunger). However, the initial interdomain orientation was lost already during the first annealing cycle. As no NOE-derived distances are available for CaM in complex with the smooth muscle myosin light chain kinase, those NOE distances measured for CaM in complex with the rabbit skeletal myosin light chain kinase (Ikura et al.) which are compatible with the crystal structure of CaM-MLCK (Meador et al.), i.e., with a violation < 1 Å, were used in the calculations. Overall, 2249 and 1631 NOE-derived distances were used for the calculations of the Ca^{2+}-CaM and CaM-MLCK ensembles. The Ca^{2+} ions were maintained at a distance of 2.4±0.1 Å from their known ligating oxygens by 22 additional restraints.

For the S2 restraints

$$\rho_{S^2}(t) = \frac{1}{N_{S^2}} \sum \left(s^{2,\exp} - s^{2,ens} \right)^2 \qquad \textbf{[6]}$$

where the sum is taken over the number N_{S^2} of S^2 restraints and $S^{2,ens}$ values were calculated as:

$$S_k^{2,ens} = \frac{3}{2(r_k^{eff})^4} \left(\sum_{i=1}^{3} \sum_{j=1}^{3} \left[\frac{1}{N_{rep}} \sum_{l=1}^{N_{rep}} r_{i,k,l} r_{j,k,l} \right]^2 - 1 \right) \qquad \textbf{[7]}$$

where $r_{i,k}$ is the ith Cartesian component of bond vector k (Best and Vendruscolo) (Lipari and Szabo). 123 and 159 S^2 order parameters determined for Ca^{2+}-CaM and CaM-MLCK, respectively, by Wand and co-workers were taken from the literature (Lee et al.) and used in the calculations.

To enable large inter-domain motions, the previously reported method (Best and Vendruscolo) (Lindorff-Larsen et al.) of restraining S^2 order parameters was significantly modified. In the procedure described here, molecular motions on the picosecond to nanosecond timescale are restrained within the molecular frame of the individual domains of CaM without altering the overall motion of the domains. At each integration step, a mass-weighted rigid body "ghost"-alignment was performed for each domain (backbone atoms), and the non-aligned coordinates of the entire protein were stored. The restraining forces were then calculated individually for each aligned domain. Finally, the forces were rotated by the transposed alignment matrices and applied to the saved non-aligned coordinates. Hence, restraining forces were only applied within the molecular frames of non-aligned individual domains ,i.e., their overall rotational and translational motion was not affected by the restraint. When the individual domains of the saved snapshots were overlaid at the end of the structure calculations as in the "ghost"-alignment procedure, the S^2 order parameters restraints were satisfied.

The structure calculations were initiated with a preparation stage followed by 10 cycles of simulated annealing. In the preparation stage, the structures were first heated and equilibrated at 300 K (α_{S^2}=1000, α_{NOE} =1000, t=100ps, units for α_{S^2} and α_{NOE} are kcal/mol and kcal/(molÅ^4), respectively). Over the following 640 ps, the force constant α_{S^2} was progressively increased to

$2.1*10^7$ and $1.0*10^8$ for Ca^{2+}-CaM and CaM-MLCK, respectively, and α_{NOE} to $1.7*10^8$ and $1.0*10^9$. Subsequently, cycles of simulated annealing were carried out in order to sample conformational space efficiently. During each annealing cycle (lasting 170 ps), the molecules were heated to 500K and then cooled to 300K. After each cycle, 15 structures were extracted at 30 ps intervals, resulting in total of 2400 conformations for further analysis.

Control Simulations

For the control simulation using NOE-derived distances only as restraints, the structure of Ca^{2+}-CaM was first heated and equilibrated at 300 K. Over the following 640 ps, the force constant α_{NOE} was progressively increased to $1.7*10^8$. Then simulated annealing cycles were carried out in order to sample conformational space efficiently. In contrast to the standard protocol, a low α_{NOE} ($1.0*10^4$) was used during the annealing cycles. In addition, only one replica could be simulated because no S^2 order parameters were used. After each of the 20 annealing cycles one structure was extracted.

For the normal mode ensemble, a normal mode calculation was carried out on an energy minimized structure of Ca^{2+}-CaM. Minimization was done by using cycles of steepest descent and adopted basis Newton-Raphson methods. Harmonic restraints were applied on the backbone atoms and were progressively reduced at each cycle. Finally the restraints were removed and the structure minimized until the energy gradient was smaller than 10^{-10} kcal/Å. Electrostatic and Lennard-Jones interactions were force switched. The CHARMM program and the CHARMM22 (Brooks et al.) parameter set were used for the energy minimization and the normal mode analysis. The structural ensemble was generated by superposition of the first 100 modes. The ensemble properties (S^2 order parameters, RDC Q-factors) were similar when the normal mode analysis was carried out on Ca^{2+}-CaM or the NTD and CTD individually.

The setup used for the restrained simulations, i.e. , Ca^{2+}-CaM solvated a 8 Å shell of TIP3 water, was taken for the first unrestrained control simulation. A soft boundary potential was used to prevent water molecules from escaping (Beglov and Roux). The Nose-Hoover temperature coupling scheme was used to keep the T= 300K. In the second unrestrained control simulation, Ca^{2+}-CaM was solvated in a 82x65x49 $Å^3$ orthorhombic box and periodic poundary conditions were applied. This unrestrained simulation was carried out at constant N, T, and P, using the Hoover temperature-pressure coupling scheme. All calculations used an atom-based truncation scheme with a list cutoff of 14 Å, a non-bond cutoff of 12 Å, and the Lennard-Jones smoothing

function initiated at 10 Å. Electrostatic and Lennard-Jones interactions were force switched. Molecular dynamics simulations used a 2 fs integration time step and SHAKE for covalent bonds involving hydrogen atoms (Ryckaert et al.). Simulations were carried out for 2 ns.

Data Analysis

Helical Axes Definition

To calculate interhelical angles and the spatial disposition of individual helices, their axes were defined as previously described (Kuboniwa et al.). The axis orientation of an α helix consisting of K residues was calculated by averaging the positions of ten consecutive backbone atoms, starting with the N atom of residue 1 through the N atom of residue 4, and connecting this average position to the average position of ten atoms starting at the C of residue K-3. Helices were defined as follows: I(6-18), II(29-38), III(45-54), IV(65-74), V(83-91), VI(102-111), VII(118-127), VIII(139-145).

Vector Geometry Mapping (VGM)

VGM is a method developed specifically to determine the conformational changes in EF-hand motifs (Yap et al.,1999). It compares the position of the exiting helix of an EF-hand with respect to its entering helix by orienting the two helices in a reference coordinate system. A reference EF-hand motif is used to define first the coordinate system. We used the EF-hand motifs of the RDC-refined solution structures of Ca^{2+}-CaM as reference conformation. Changes in the relative orientations of helices in the query EF-hand are then reported as angles θ, ϕ, and ω within the coordinate system of the reference. θ is the angle between the exiting helix of the query EF-hand and the Z-axis, ϕ is the angle between this helix and the x-axis, and ω is the counterclockwise rotation of the helix about its long axis with respect to the exiting helix of the reference.

Distance Difference (DD) Analysis

The DD (Nelson and Chazin) is defined as the difference between the distance of a given atom pair in one structure (Ca^{2+}-CaM) and the distance of the same atom pair in another structure (CaM-MLCK):

$$DD_{ij} = d_{ij}(free) - d_{ij}(bound) \quad [8]$$

where d_{ij} is the distance between atom i and j. For this analysis only Cα-atoms were used. To get a single value for a whole structure or segments of it, we defined $RMSDD_{ij}$ as:

$$RMSDD_{ij} = \sqrt{\sum^{N} \frac{(dd_{ij}(free) - dd_{ij}(bound))^2}{N}} \qquad [9]$$

where N is the number of possible atom pairs in the entire protein or the selected segment.

Analysis of Interdomain Motions

To calculate the S^2global, the structures of the Ca^{2+}-CaM ensemble were first aligned on the entire CTD, and then the normalized vector connecting the residue at the centre of the flexible linker (residue 79) with the centre of mass of the NTD was used in Eq. **7**.

We analysed the global orientations of the two CaM domains in the Ca^{2+}-CaM state in several ways. First, we monitored the distribution of the position of the axis of helix IV whilst keeping helix V fixed (Figures 6A and 6B). This analysis was repeated by aligning the entire CTD of the different structures instead of just the helix V regions (Figure S8). This type of analysis allows monitoring very accurately the getting together of the two domains and was subsequently used to determine how compaction of CaM affects internal motion (Figure 6E–6H). However, to fully assess the motions of the two domains with respect to each other, Euler angles defining the orientations of the NTD with respect to the CTD were calculated. To do so, an orthogonal axis system was first defined. The axis of helix 4 was defined as γ-axis, the β-axis is perpendicular to this axis in the plane defined by the axes of helix 4 and helix 3, and the α-axis is perpendicular to β and γ. After alignment of the CTD, Euler angles were determined which allow reproducing the orientation of NTD in the CaM-MLCK complex.

Supplemental References

Aoyagi, M., Arvai, A.S., Tainer, J.A., and Getzoff, E.D. (2003). Structural basis for endothelial nitric oxide synthase binding to calmodulin. Embo J *22*, 766-775.

Beglov, D. and Roux, B. (1994). Finite representation of an infinite bulk system: solvent boundary potential for computer simulations. J. Chem. Phys. 100, 9050-9063.

Bentrop, D., Bertini, I., Cremonini, M.A., Forsen, S., Luchinat, C., and Malmendal, A. (1997). Solution structure of the paramagnetic complex of the N-terminal domain of calmodulin with two Ce3+ ions by 1H NMR. Biochemistry *36*, 11605-11618.

Berman, H.M., Westbrook, J., Feng, Z., Gilliland, G., Bhat, T.N., Weissig, H., Shindyalov, I.N., and Bourne, P.E. (2000). The Protein Data Bank. Nucleic Acids Res *28*, 235-242.

Best, R.B., Clarke, J., and Karplus, M. (2005). What contributions to protein side-chain dynamics are probed by NMR experiments? A molecular dynamics simulation analysis. J Mol Biol *349*, 185-203.

Best, R.B., and Vendruscolo, M. (2004). Determination of protein structures consistent with NMR order parameters. J Am Chem Soc *126*, 8090-8091.

Brooks, B. R., Bruccoleri, R. E., Olafson, B. D., States, D. J., Swaminathan, S. and Karplus, M. (1983). CHARMM: a program for macromolecular energy, minimization, and dynamics calculations. J. Comput. Chem. 4, 187-217.

Chou, J.J., Li, S., Klee, C.B., and Bax, A. (2001). Solution structure of Ca(2+)-calmodulin reveals flexible hand-like properties of its domains. Nat Struct Biol *8*, 990-997.

Clapperton, J.A., Martin, S.R., Smerdon, S.J., Gamblin, S.J., and Bayley, P.M. (2002). Structure of the complex of calmodulin with the target sequence of calmodulin-dependent protein kinase I: studies of the kinase activation mechanism. Biochemistry *41*, 14669-14679.

Contessa, G.M., Orsale, M., Melino, S., Torre, V., Paci, M., Desideri, A., and Cicero, D.O. (2005). Structure of calmodulin complexed with an olfactory CNG channel fragment and role of the

central linker: residual dipolar couplings to evaluate calmodulin binding modes outside the kinase family. J Biomol NMR *31*, 185-199.

Fallon, J.L., Halling, D.B., Hamilton, S.L., and Quiocho, F.A. (2005). Structure of calmodulin bound to the hydrophobic IQ domain of the cardiac Ca(v)1.2 calcium channel. Structure *13*, 1881-1886.

Finn, B.E., Evenas, J., Drakenberg, T., Waltho, J.P., Thulin, E., and Forsen, S. (1995). Calcium-induced structural changes and domain autonomy in calmodulin. Nat Struct Biol *2*, 777-783.

Ikura, M., Clore, G.M., Gronenborn, A.M., Zhu, G., Klee, C.B., and Bax, A. (1992). Solution structure of a calmodulin-target peptide complex by multidimensional NMR. Science *256*, 632-638.

Kuboniwa, H., Tjandra, N., Grzesiek, S., Ren, H., Klee, C.B., and Bax, A. (1995). Solution structure of calcium-free calmodulin. Nat Struct Biol *2*, 768-776.

Lee, A.L., Kinnear, S.A., and Wand, A.J. (2000). Redistribution and loss of side chain entropy upon formation of a calmodulin-peptide complex. Nat Struct Biol *7*, 72-77.

Lindorff-Larsen, K., Best, R.B., Depristo, M.A., Dobson, C.M., and Vendruscolo, M. (2005). Simultaneous determination of protein structure and dynamics. Nature *433*, 128-132.

Lipari, G. and Szabo, A. (1982) Model-free aaproach to the interpretation of NMR relaxation in Macromolecules. J. Am. Chem. Soc. *104*, 4546-4559.

Meador, W.E., Means, A.R., and Quiocho, F.A. (1993). Modulation of calmodulin plasticity in molecular recognition on the basis of x-ray structures. Science *262*, 1718-1721.

Nelson, M.R., and Chazin, W.J. (1998). An interaction-based analysis of calcium-induced conformational changes in Ca2+ sensor proteins. Protein Sci *7*, 270-282.

Osawa, M., Tokumitsu, H., Swindells, M.B., Kurihara, H., Orita, M., Shibanuma, T., Furuya, T., and Ikura, M. (1999). A novel target recognition revealed by calmodulin in complex with Ca2+-calmodulin-dependent kinase kinase. Nat Struct Biol *6*, 819-824.

Paci, E., Vendruscolo, M., Dobson, C.M., and Karplus, M. (2002). Determination of a transition state at atomic resolution from protein engineering data. J Mol Biol *324*, 151-163.

Richter, B., Gsponer, J., Varnai, P., Salvatella, X., and Vendruscolo, M. (2007). The MUMO (minimal under-restraining minimal over-restraining) method for the determination of native state ensembles of proteins. J Biomol NMR *37*, 117-135.

Ryckaert, J. P., Ciccotti, G. and Berendsen, H. J. C. (1977). Numerical integration of the Cartesian equation of motion of a system with constraints: Molecular dynamics of n-alkanes. J. Comp. Phys. 23, 327-341.

Schumacher, M.A., Rivard, A.F., Bachinger, H.P., and Adelman, J.P. (2001). Structure of the gating domain of a Ca2+-activated K+ channel complexed with Ca2+/calmodulin. Nature *410*, 1120-1124.

Shen, Y., Lee, Y.S., Soelaiman, S., Bergson, P., Lu, D., Chen, A., Beckingham, K., Grabarek, Z., Mrksich, M., and Tang, W.J. (2002). Physiological calcium concentrations regulate calmodulin binding and catalysis of adenylyl cyclase exotoxins. Embo J *21*, 6721-6732.

Wall, M.E., Clarage, J.B., and Phillips, G.N. (1997). Motions of calmodulin characterized using both Bragg and diffuse X-ray scattering. Structure *5*, 1599-1612.

Wilson, M.A., and Brunger, A.T. (2000). The 1.0 A crystal structure of Ca(2+)-bound calmodulin: an analysis of disorder and implications for functionally relevant plasticity. J Mol Biol *301*, 1237-1256.

Yap, K.L., Ames, J.B., Swindells, M.B., and Ikura, M. (1999). Diversity of conformational states and changes within the EF-hand protein superfamily. Proteins *37*, 499-507.

Yap, K.L., Yuan, T., Mal, T.K., Vogel, H.J., and Ikura, M. (2003). Structural basis for simultaneous binding of two carboxy-terminal peptides of plant glutamate decarboxylase to calmodulin. J Mol Biol *328*, 193-204.

Table S1. Comparison of Experimental and Calculated Backbone S^2 Order Parameters in Different Structural Ensembles of Ca^{2+}-CaM

	NTD	*CTD*
Experimental	0.91[a]	0.85[a]
Restrained simulation (NOE + S^2)	0.88 (0.96)[b]	0.82 (0.95)
Restrained simulation (NOE only)	0.84 (0.70)	0.73 (0.63)
Normal-mode	0.96 (0.34)	0.97 (0.15)
MD simulation with water shell	0.80 (0.52)	0.67 (0.25)
MD simulation with water box	0.86 (0.54)	0.76 (0.47)

[a]Average backbone S^2 order parameters in the two domains of Ca^{2+}-CaM. [b]Correlations between experimental and calculated S^2 order parameters are given in parenthesis.

Table S2. $RMSDD_{ij}$ between Structural Segments of the Ca^{2+}-CaM Ensemble and the CaM-MLCK Ensemble

Segment (helices)	*$RMSDD_{ij}$ (Å)*
6-38 (I-II)	1.21
**29-54 (II-III)**	**0.77**
45-78 (III-IV)	2.11
80-111 (V-VI)	1.15
**101-130 (VI-VII)**	**0.94**
118-145 (VII-VIII)	1.12

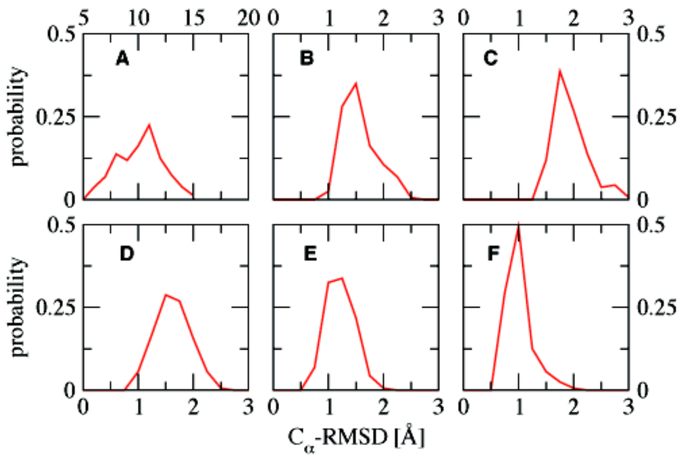

Figure S1. Distribution of Root Mean Square Deviations between the Calculated Ensembles and the RDC-Refined (Chou et al.) and X-ray Structures of Ca^{2+}-CaM (Wilson and Brunger) and CaM-MLCK (Meador et al.), Respectively

A) RMSD of Ca^{2+}-CaM with respect to the X-ray structure. B) RMSD of NTD of Ca^{2+}-CaM with respect to the RDC-refined structure. C) RMSD of CTD of Ca^{2+}-CaM with respect to the RDC-refined structure D) RMSD of CaM-MLCK with respect to the X-ray structure. E) RMSD of NTD of CaM-MLCK with respect to the X-ray structure. F) RMSD of CTD of CaM-MLCK with respect to the X-ray structure.

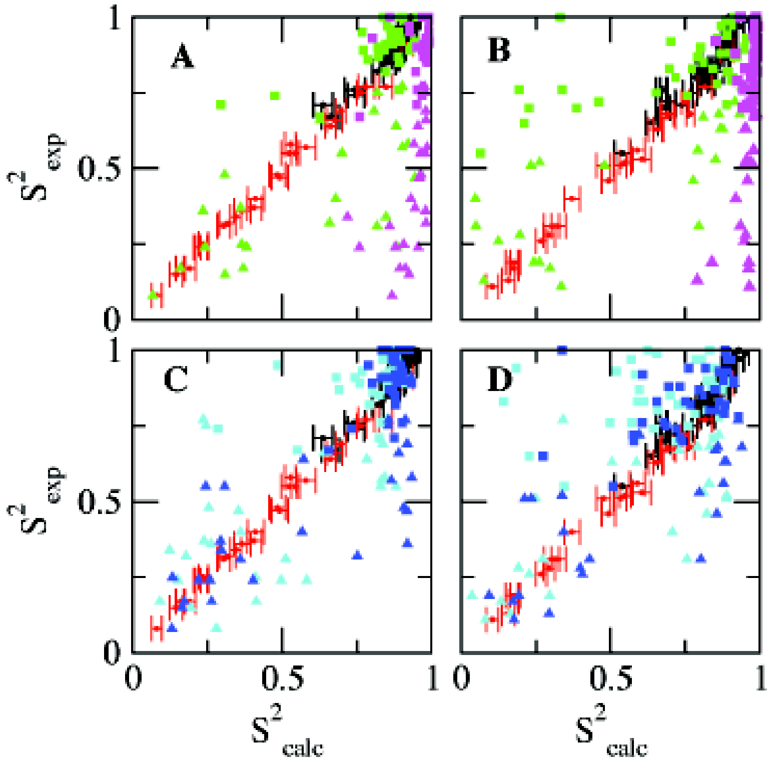

Figure S2. Comparison of Experimental and Calculated S^2 Order Parameters in the Different Ensembles of Ca^{2+}-CaM that We Calculated

S^2 order parameters of the NTD are shown in A and C, those of the CTD are shown in B and D. In all panels, the backbone and side chain S^2 order parameters of the Ca^{2+}-CaM ensemble generated by molecular dynamics simulations with NOE and S^2 order parameter restraints are shown in black and red, respectively. A-B) Backbone (squares) and side chain (triangles) S^2 order parameters in the ensembles determined with NOE restraints only (green), and by the superposition of the first 100 normal modes (magenta). C-D) Backbone (squares) and side chain (triangles) S^2 order parameters in the ensembles determined by classical molecular dynamics simulations using either a water shell (cyan) or a water box (blue).

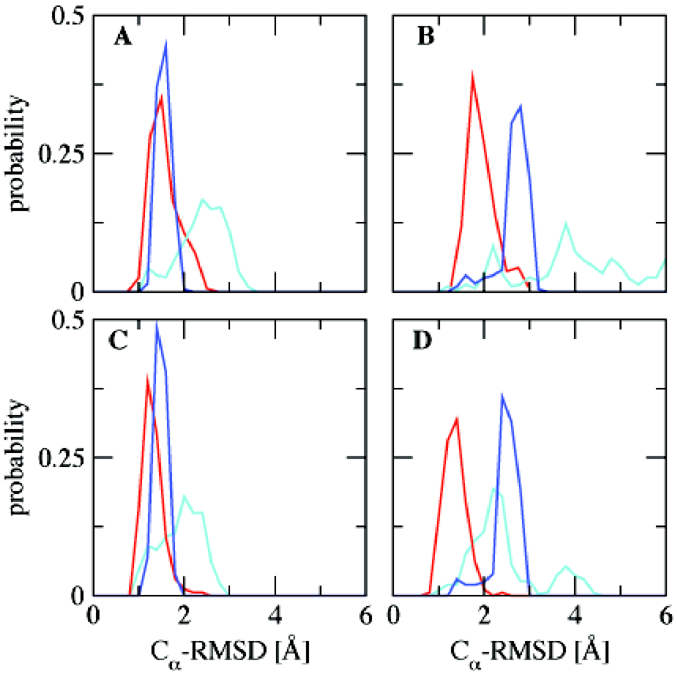

Figure S3. Distribution of Root Mean Square Deviations between Different Calculated Ensembles of Ca^{2+}-CaM and the RDC-Refined Structures of Ca^{2+}-CaM (Chou et al.)

RMSDs of the ensembles generated by molecular dynamics simulations with NOE and S^2 order parameter restraints, classical molecular dynamics simulations of Ca^{2+}-CaM in a water shell and classical molecular dynamics simulations of Ca^{2+}-CaM in a water box are shown in red, cyan and blue, respectively. A) RMSD of NTD of Ca^{2+}-CaM (backbone atoms of residues 4-78) with respect to the RDC-refined structure. B) RMSD of CTD of Ca^{2+}-CaM (backbone atoms of residues 80-148) with respect to the RDC-refined structure. C) RMSD of NTD of Ca^{2+}-CaM (backbone atoms of residues 9-73) with respect to the RDC-refined structure. B) RMSD of CTD of Ca^{2+}-CaM (backbone atoms of residues 85-145) with respect to the RDC-refined structure.

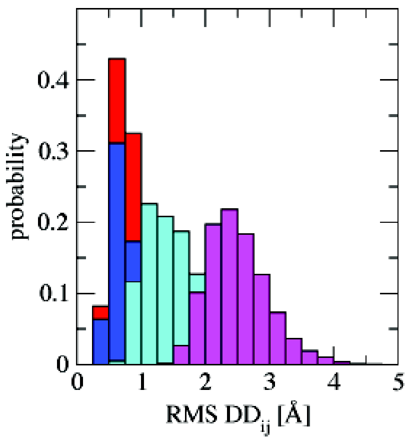

Figure S4. Distribution of Root Mean Square Distance Differences (RMSDD) between the Structures of the Ca^{2+}-CaM and CaM-MLCK Ensembles

RMSDD$_{ij}$ of atom pairs in the segment 29-54 are shown in blue, those of the atom pairs in the remaining parts of the NTD (residues 5-28 and 55-78) are shown in magenta. RMSDD$_{ij}$ of atom pairs in the segment 101-130 are shown in red, those of the atom pairs in the remaining parts of the CTD (residues 80-100 and 131-146) are shown in cyan.

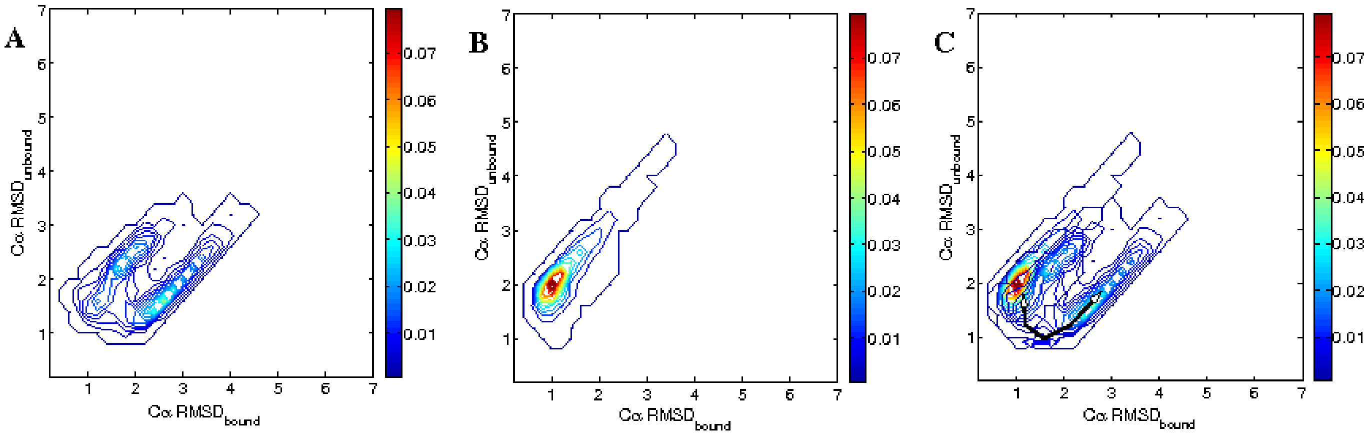

Figure S5. Comparison of Structural Properties in the Ca^{2+}-CaM and CaM-MLCK Ensembles

A) RMSD of helices V and VIII with respect to the RDC-refined solution structure of Ca^{2+}-CaM (Cα-RMSD$_{unbound}$) and the crystal structure of CaM-MLCK (Cα-RMSD$_{bound}$) in the Ca^{2+}-CaM ensemble. B) RMSD of helices V and VIII with respect to the RDC-refined solution structure of Ca^{2+}-CaM (Cα-RMSD$_{unbound}$) and the crystal structure of CaM-MLCK (Cα-RMSD$_{bound}$) in the CaM-MLCK ensemble. C) Superposition of A and B. Projection of the first (black) and fourth (blue) modes of motion in the CTD on Cα-RMSD$_{unbound}$ and Cα-RMSD$_{bound}$, respectively.

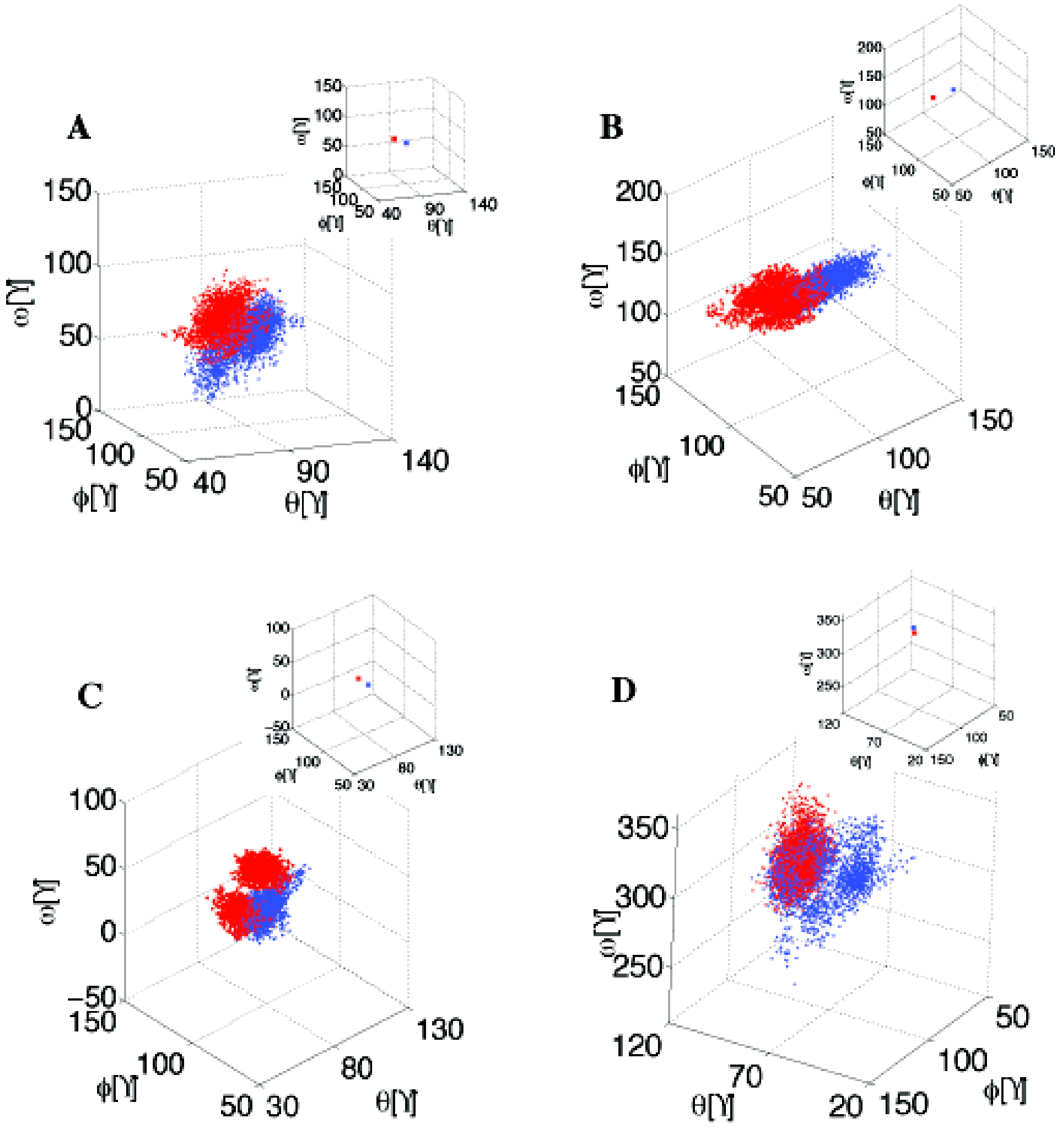

Figure S6. Inter-Helical Angles Are Commonly Used to Describe the Conformational Changes in CaM

Since one angle is not sufficient to determine the exact position of two helices with respect to each other, we analysed the position of the helices in each EF hand in further detail with the help of the Vector Geometry Mapping (VGM) method (Yap et al.,1999) (see Supplemental Experimental Procedures). The results of the VGM analysis are shown in this figure. Distribution of the θ, ϕ, and ω angles that describe the tertiary structure of each EF hand in the ensembles of Ca^{2+}- CaM (red) and CaM-MLCK (blue), respectively. The first and second EF hand in the NTD (helices I-II and

III-IV, respectively) are shown in A and B, respectively. The first and second EF hand in the CTD (helices V-VI and VII-VIII, respectively) are shown in C and D, respectively. The θ, ϕ, and ω angles observed in the RDC-refined solution structure of Ca^{2+}- CaM (red) and the X-ray structure CaM-MLCK (blue) are shown in the inlay. Overall we find a significant overlap between the Ca^{2+}-CaM and CaM-MLCK states of CaM with respect to the tertiary structure of their EF-hands; this overlap is more pronounced in the CTD.

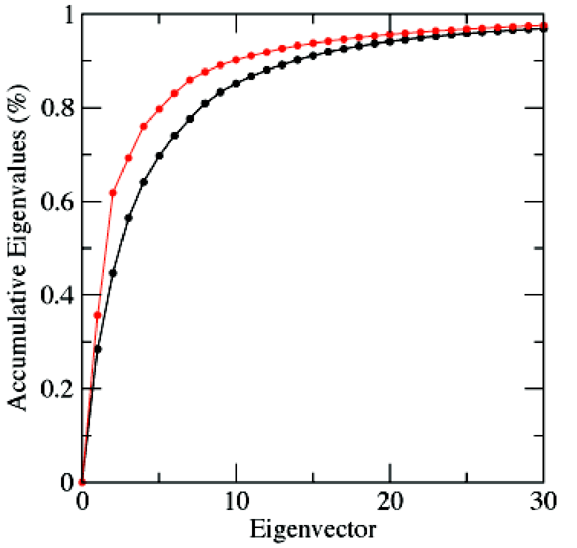

Figure S7. Cumulative Sum of Normalised Eigenvalues

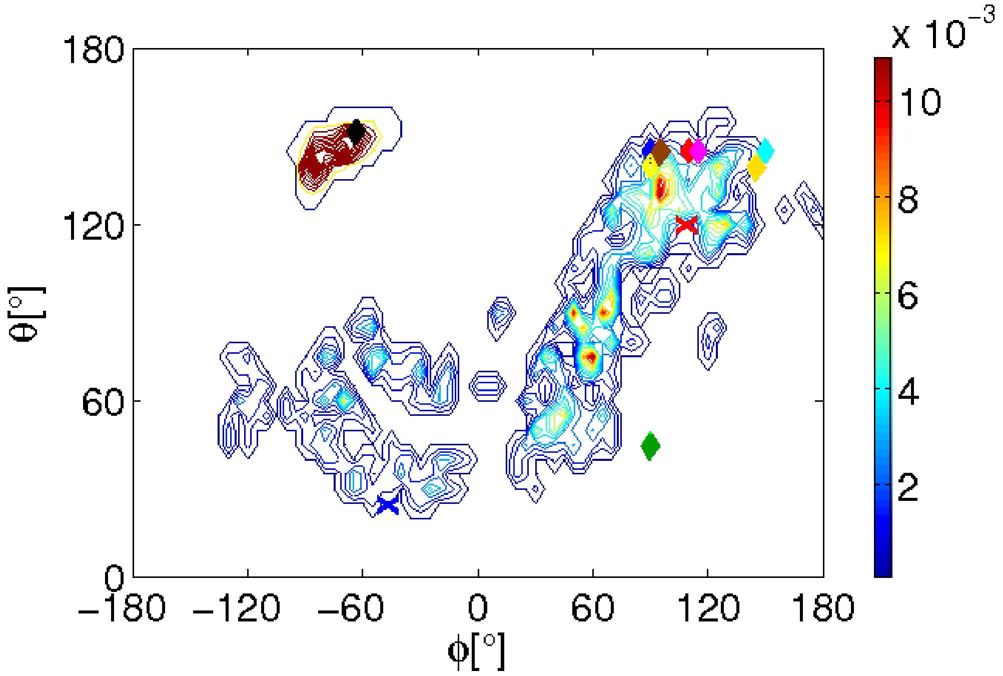

Figure S8. Analysis of the Interdomain Motion in the Ca^{2+}-CaM Ensemble

Distribution of the axis of helices IV and V in the Ca^{2+}-CaM ensemble in polar coordinates. Structures were aligned on the entire CTD. The position of the helix IV and V in the crystal

structure of CaM-MLCK are shown as a red and black diamond, respectively. The position of the axis of helix IV in other complexes of CaM are indicated by additional diamonds (fully Ca^{2+} loaded) and crosses (partially loaded); the complexes of CaM with CaMKK (1CKK) (Osawa et al.), CAMKI (1MXE.pdb) (Clapperton et al.), CaMKII (1CM1.pdb) (Wall et al.), NO synthase (1NIW.pdb) (Aoyagi et al.), glutamate decarboxylase (1NWD.pdb) (Yap et al., 2003), olfactory CNG channel (1SY9.pdb) (Contessa et al.) and voltage-gated calcium channel (2F3Y.pdb) (Fallon et al.) are indicated as magenta, yellow, blue, cyan, green, brown and orange diamonds, respectively (fully Ca2+ loaded); the complexes of CaM with the Ca2+-activated K+ channel (1G4Y.pdb) (Schumacher et al.) and the adenylyl cyclase domain of the anthrax edema factor (1LVC.pdb) (Shen et al.) as red and blue crosses, respectively. The positions of the axis of helix V in all these complexes cluster around the black diamond and are not shown for the sake of clarity.

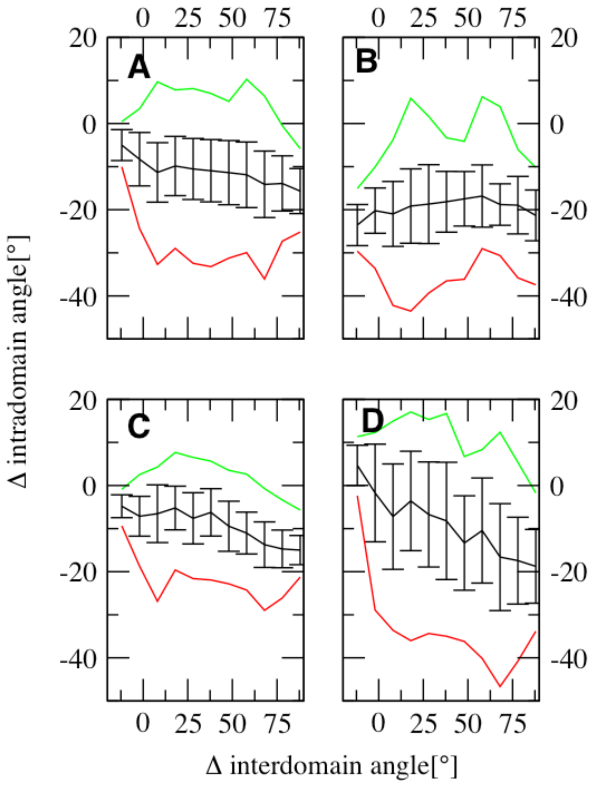

Figure S9. Correlation between Intra- and Interdomain Motions in the Ca^{2+}- CaM Ensemble
A-D) Deviations of inter-helical angles in the Ca^{2+}-CaM ensemble from that found in the complex as a function of the deviation of the interdomain angle between helices IV and V from that in CaM-MLCK. Inter-helical angles I-II,III-IV, V-VI, and VII-VIII are shown in A, B, C,and D, respectively.
